# Quantitative bioluminescence tomography using spectral derivative data

**DOI:** 10.1364/BOE.9.004163

**Published:** 2018-08-09

**Authors:** Hamid Dehghani, James A. Guggenheim, Shelley L. Taylor, Xiangkun Xu, Ken Kang-Hsin Wang

**Affiliations:** 1 School of Computer Science, University of Birmingham, Birmingham, B15 2TT, UK; 2 Department of Medical Physics & Biomedical Engineering, University College London, London, UK; 3 Department of Radiation Oncology and Molecular Radiation Sciences, Johns Hopkins University, Baltimore, MD, USA

**Keywords:** (170.3010) Image reconstruction techniques, (170.6280) Spectroscopy, fluorescence and luminescence, (170.6960) Tomography, (100.3190) Inverse problems

## Abstract

Bioluminescence imaging (BLI) is a non-contact, optical imaging technique based on measurement of emitted light due to an internal source, which is then often directly related to cellular activity. It is widely used in pre-clinical small animal imaging studies to assess the progression of diseases such as cancer, aiding in the development of new treatments and therapies. For many applications, the quantitative assessment of accurate cellular activity and spatial distribution is desirable as it would enable direct monitoring for prognostic evaluation. This requires quantitative spatially-resolved measurements of bioluminescence source strength inside the animal to be obtained from BLI images. This is the goal of bioluminescence tomography (BLT) in which a model of light propagation through tissue is combined with an optimization algorithm to reconstruct a map of the underlying source distribution. As most models consider only the propagation of light from internal sources to the animal skin surface, an additional challenge is accounting for the light propagation from the skin to the optical detector (e.g. camera). Existing approaches typically use a model of the imaging system optics (e.g. ray-tracing, analytical optical models) or approximate corrections derived from calibration measurements. However, these approaches are typically computationally intensive or of limited accuracy. In this work, a new approach is presented in which, rather than directly using BLI images acquired at several wavelengths, the spectral derivative of that data (difference of BLI images at adjacent wavelengths) is used in BLT. As light at similar wavelengths encounters a near-identical system response (path through the optics etc.) this eliminates the need for additional corrections or system models. This approach is applied to BLT with simulated and experimental phantom data and shown that the error in reconstructed source intensity is reduced from 49% to 4%. Qualitatively, the accuracy of source localization is improved in both simulated and experimental data, as compared to reconstruction using the standard approach. The outlined algorithm can widely be adapted to all commercial systems without any further technological modifications.

## 1. Introduction

Bioluminescence Imaging (BLI) is a highly sensitive and non-invasive pre-clinical imaging technique based on the detection of visible and near-infrared light produced by, for example, luciferase-catalyzed reactions (bioluminescence) [1]. This method allows for the non-invasive detection and visualization in 2D of functional activity within intact living animals and is becoming widespread due to the prognostic insights it can provide into established model of disease. However, the quality and quantitative accuracy of the information that BLI can provide is tempered by the limitations of the 2D planar information obtained.

BLI allows non-invasive imaging of whole organisms, whereby bioluminescent emissions at a given wavelength (typically 500 – 650 nm) are recorded and maps of ‘molecular beacons’ are used to infer cellular activity and spatial distribution [2]. It offers near real-time monitoring of spatial and temporal progression of molecular processes in the same animal, as opposed to euthanizing a cohort of animals. One major advantage of such a technique is the possibility of acquiring a whole-body image within one exposure cycle (typically in order of minutes), which can significantly shorten the subject study time. However, accurate quantification of the spatial location and intensity of the light (which is then often used to infer the cellular activity) cannot be established due to several factors, including the often limited number of wavelengths measured and inaccurate mapping of the measured signal on the 2D detector (often a CCD) onto the 3D surface of the animal (free-space light propagation mapping) as well as the unknown underlying and spectrally varying tissue optical properties [3]. These are the primary reasons that almost all commercially available systems only use ‘topographic’ (2D surface) single wavelength pseudo-mapping of bioluminescence (that is an acquired image of the bioluminescence signal superimposed onto a textured image of the animal).

To allow a more quantitative analysis from BLI, methods that allow the recovery of spatially resolved tomographic maps of bioluminescence source location and intensity have been proposed to allow Bioluminescence Tomography (BLT) [4–8]. The basic idea is to employ a ‘forward’ model of light propagation through tissue to the skin surface in conjunction with an optimization algorithm to reconstruct the underlying bioluminescence source distribution. For single-wavelength data, this inverse problem is highly non-unique [9], i.e. identical measurements are produced by many different light source distributions. However, as bioluminescent sources have broadband emission spectra (e.g. 500-650 nm for firefly Luciferase/luciferin), spectrally-resolved detection schemes (e.g. bandpass filters) can be used to measure the emission due to the bioluminescence activity at the surface of the tissue at several wavelengths in order to reduce the non-uniqueness.

To date, all reported BLT reconstruction algorithms have either used a priori information to constrain the solution and keep the computation time low [8], or analytical solutions which limit the problem to a homogenous tissue volume with regular shapes [6]. To achieve quantitatively accurate recovery of sources in a complex and heterogeneous model, there is a need to derive and construct a robust method that can accurately model light propagation in heterogeneous and complex tissue, using for example, the Finite Element Method (FEM) of light propagation in tissue [10]. 3D modeling and reconstruction algorithm as applied to multi-wavelength 3D BLT image reconstruction have widely been developed and to improve the image recovery accuracy and computation time, we have previously reported the reciprocity approach [5], which is similar to that used in Diffuse Optical Tomography (DOT) and Fluorescence DOT [11]. Furthermore, it has also been demonstrated that through accounting and modeling for the system characteristics such as filter bandwidth the accuracy of the recovered bioluminescence distribution can be improved [12].

In all cases for tomographic reconstruction of bioluminescence source distribution, the data used for the parameter recovery has been based on ‘intensity’ of the measured bioluminescence, rather than the more conventionally used ‘logarithm of intensity’ as in DOT, where the ‘logarithm of intensity’ is utilized to overcome the large dynamic range of the measured data and to improve sensitivity of the problem [13]. However, when considering Fluorescence Tomography, it has been demonstrated that the use of ‘normalized born-ratio’ where the emission data is normalized by the excitation data to account for the unknown tissue attenuation has shown to be more robust for parameter recovery [14, 15]. To date however, the utilization of neither the ‘logarithm of intensity’, nor the ratio-metric data has been investigated in BLT.

The underlying optical properties of the animals being imaged are determined by the concentrations of different tissue chromophores such as oxy and deoxy hemoglobin, water and fat, as well as the scattering properties of tissue. Therefore, a change in the underlying ‘spectrally’ varying optical properties will lead to a change in attenuation of the tissue, resulting in a different surface fluence arising from an internal bioluminescent source, which can be overcome through use of a dual-modality DOT and BLT [16]. Additionally, given that all bioluminescence imaging systems are based on a non-contact configuration, it has been demonstrated that a change in position of the animal can result in a different measured signal, which can however be accounted for by modelling the propagation of light from the surface of the animal to the CCD using a free-space model [17]. This free-space model accounts for the propagation of light from the surface of the imaging subject to the CCD calculating a mapping matrix which describes the contribution of each point on the surface of the imaging subject to each pixel in the CCD. Inverting this relationship enables CCD data to be mapped back on to the surface of the imaging subject, determining true surface fluence values which are independent of the position of the imaging subject. However, this requires an accurate knowledge about the 3D surface topology of the domain being imaged, which may not always be available and therefore an image reconstruction which is independent of the domain geometry and positioning would be advantageous.

In this work, we present the importance of the domain geometry and animal position on the measured bioluminescence fluence and demonstrate that unless accounted for, it will lead to erroneous parameter recovery. A new image reconstruction algorithm is outlined and validated which is based on the spectral derivative of the measured spectral data. Through both simulations and phantom data measurements, the benefits of using ‘logarithm of intensity’ for image reconstruction which allows for spectral derivate data to be utilized in BLT is highlighted which is shown to overcome this so called ‘free-space’ light propagation error.

## 2. Methods and results

### 2.1 Intensity variation due to surface geometry

An experiment was undertaken to demonstrate the impact of the imaging system and animal position on BLI measurements in a commonly utilized commercial scanner. Images were acquired using an IVIS Spectrum (Perkin Elmer) with an “open” filter using a 4 pixel binning (providing images of 256 by 256 resolution). The imaged object was a cylindrical phantom (Biomimic, INO, Quebec, Canada) of dimensions Ø25 x 50mm (~mouse-sized). The phantom is made of a solid plastic with homogeneous spectrally-varying optical absorption and scattering properties that have been characterized within the range of 500 to 850nm in terms of the absorption coefficient, μ_a_ = [0.007 – 0.12]mm^−1^, and the reduced scattering coefficient, μ_s’_ = [1.63 – 1.79]mm^−1^ [18]. Within the phantom body there are two tunnels with a diameter of 6mm at depths of 5mm and 15mm in which rods (cylindrical inclusions) can be inserted to match the background, effectively creating a solid homogeneous cylinder. In this study, bioluminescence is achieved by placing a light source half way along a tunnel enclosed between two rods of background matching material.

**Fig. 1:**
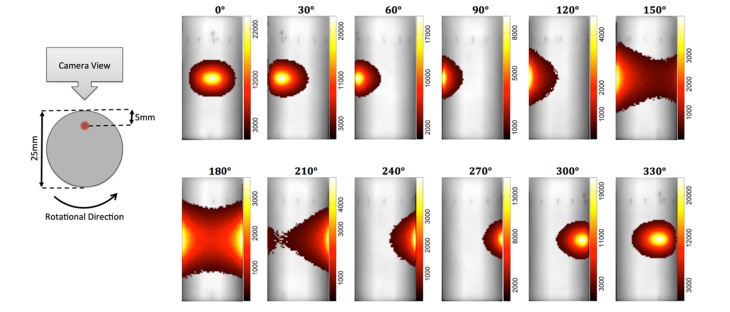
Schematic of the imaging protocol and phantom setup and the total photon count images from the source at 5 mm depth with varying rotational angles.

To mimic in vivo bioluminescence experiments, a small self-sustained tritium-based light source (Trigalight Orange III; MB-Microtec, Niederwangen, Switzerland) was used as an artificial bioluminescence source which is 0.9 × 2.5mm in size. The emission spectrum of the tritium-based light source is a Gaussian-like curve with a central peak at 606nm and a full-width-half-maximum of approximately 100nm, meaning that it is similar to the spectral output of a bioluminescent reporter. The light source was placed at a depth of 5 mm inside the cylinder phantom which was then rotated from 0 – 330 degrees in steps of 30 degrees in order, such that the effective target source location was kept at a constant depth by varying angles with respect to the imaging camera, Fig. 1

.

**Fig. 2:**
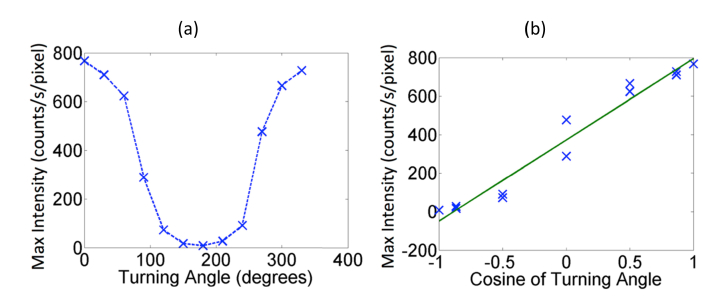
Measured intensity data from the cylindrical phantom as function of (a) angle and (b) the cosine of turning angle.

Captured images were exported from the IVIS in Tagged Image File Format and the detected photon intensity as a function varying angles are shown in Fig. 1. Several features are apparent from the captured data, namely that the detected maximum photon count is reducing as a function of increasing angle away from the camera and that the apparent size of the source is also increasing. In order to better demonstrate the variation of the detected photon count as a function of angular rotation of the phantom, the detected maximum intensity of the signal for each angular rotation is plotted in Fig. 2(a)

with respect to the view point angle of the camera. It is seen that the measured maximum intensity is symmetric as a function of the angular rotation and that the measured intensity has a non-linear decaying function as the apparent angle with respect to the camera changes.

Given that the photon exiting the surface of the phantom is Lambertian (i.e. the intensity is observed from a diffusely reflecting surface), maximum number of photons would be exiting the phantom at an angle normal to the surface. Therefore, the detected photons by the camera will be a function of the cosine of the angle between the normal of phantom surface point and normal of the focal plane of the camera, as well as the associated distance to the camera. To demonstrate this, the same maximum photon count shown in Fig. 2(a) is also plotted as function of this cosine angle and shown in Fig. 2(b). This demonstrates that there is a linear dependency which must be accounted for to ensure quantitative analysis. It should also be highlighted that for angular rotations between 90 and 270 degrees, as the surface normal at the point of maximum intensity is not visible to the camera, there will also exist variations in intensity due to optical attenuation of the signal, which accounts for the larger deviations from the expected linear relationship shown in Fig. 2(b) for these angles. In a system, where views from wider angles are possible, due to for example use of mirrors, we have demonstrated that this variation in intensity does follow a Lambertian nature [17].

### 2.2 Image reconstruction: the conventional approach

Given the underlying spectrally varying optical properties of the object being imaged, the aim of the image reconstruction is the recovery of the unknown bioluminescence source at each point within the volume using measurements of bioluminescence light from the tissue surface. This can be represented as a set of linear equations of the form:

$$A_{\lambda }x=b_{\lambda }$$ (1)

where *A* is a sensitivity matrix for a given wavelength *λ* calculated using the Adjoint model, which relates changes in the measured boundary data *b* (at the surface) at the same wavelength with respect to the spatially varying bioluminescence distribution *x*. As the imaging problem is known to be non-unique, it has been shown that using measurements at multiple wavelengths can help overcome this issue due to the unique spectrally varying attenuation of biological tissue. Therefore, typically *b* is the measured intensity at a number of wavelengths, spanning the emission spectrum of the bioluminescence source (typically between 550 – 650 nm) and therefore *A* is also calculated for the same wavelengths. Assuming a total of 4 wavelengths Eq. (1) becomes:

$$\left[\begin{array}{c} \begin{array}{c} A_{\lambda 1}\\ A_{\lambda 2} \end{array}\\ A_{\lambda 3}\\ A_{\lambda 4} \end{array}\right]x=\left[\begin{array}{c} \begin{array}{c} b_{\lambda 1}\\ b_{\lambda 2} \end{array}\\ b_{\lambda 3}\\ b_{\lambda 4} \end{array}\right]$$ (2)

which then can be solved for *x*. In this work a compressive sensing conjugate gradient (CSCG) based method is utilized, which assumes a sparse source distribution, as is typically the case when studying the growth and kinetics of localized cancerous tumors, which has been shown to reduce the inherent ill-posed nature of BLT reconstructions [19].

Consider the case where there exists an angular dependent offset for each measurement b, as demonstrated in in Fig. 2. Equation (2) then becomes:

$$A_{\lambda }x=b_{\lambda }n$$ (3)

where *n* is a measurement point specific angular dependent offset, which is assumed to be spectrally invariant. This assumption is valid, as the path of light at these wavelengths is only dependent on the external geometry of the medium as well as the intrinsic properties of the lens and location of the imaging camera. It is therefore apparent that assuming the offset *n* can be defined by the relationship shown in Fig. 2(b), then the recovered maps of the spatial distribution of the bioluminescence source will be heavily corrupted through the inversion step of the image reconstruction.

### 2.3 Image reconstruction: the spectral derivative approach

The use of the spectral derivative of the measured data has been previously shown to overcome the effects of unknown source and detector noise (coupling coefficients), as applied in Near Infrared Spectroscopy (NIRS) [20]. Due to the typically large dynamic range of NIRS data, the logarithm of the measured intensity is used which then allows the cancellation of these unknown and yet spectrally constant coupling coefficients. In the case of BLT, Eq. (3) needs to be modified such that the right-hand side of the equation is dependent on the logarithm of the measured intensity. Given that:

$$\log b=\frac{\log b}{b}b$$ (4)

then both sides of Eq. (3) can be multiplied by $\frac{\log(b_{\lambda }n)}{b_{\lambda }n}$ to give:

$$\frac{\log(b_{\lambda }n)}{b_{\lambda }n}A_{\lambda }x=\log(b_{\lambda }n)$$ (5)

The spectral derivative method relies on the fact that rather than using data at each given wavelength, as defined in Eq. (2), the difference of data between each nearest wavelength is instead utilized. By this definition, for two neighboring wavelengths *λ_i_* and *λ_i + 1_*:

$$\frac{\log(b_{\lambda i}n)}{b_{\lambda i}n}A_{\lambda i}x=\log(b_{\lambda i}n)$$ (6)

$$\frac{\log(b_{\lambda i+1}n)}{b_{\lambda i+1}n}A_{\lambda i+1}x=\log(b_{\lambda i+1}n)$$ (7)

and subtracting Eq. (7) from Eq. (6):

$$\left[\frac{\log(b_{\lambda i}n)}{b_{\lambda i}n}A_{\lambda i}-\frac{\log(b_{\lambda i+1}n)}{b_{\lambda i+1}n}A_{\lambda i+1}\right]x=\log(\frac{b_{\lambda i}}{b_{\lambda i+1}})$$ (8)

where any offset in the right-hand side of the equation, due to geometrical shape of the object being imaged or camera properties (intrinsic and extrinsic) that are spectrally independent and constant, are cancelled from the right-hand side of the equation. Although the sensitivity matrix *A* is normalized by the data containing *n*, this is not consequential as the normalized measurement (right-hand-side of Eq. (8)) no longer depends on this unknown parameter *n*. It is also worth highlighting that this normalization of the A matrix, whereby the relationship is transformed to its logarithm is also known and shown to be less ill-posed than its original intensity one [13].

### 2.4 Numerical simulations

To demonstrate the effects of the uncalibrated errors due to geometrical shape of the object being imaged or camera properties on the image reconstruction using the conventional method (Eq. (2)) and the advantage of using the spectral derivative method based on ‘logarithm of intensity’ data (Eq. (8)), 2D simulations are performed. All simulations are performed in NIRFAST which is an open-source Finite Element model-based image reconstruction for diffuse optics and molecular imaging (www.nirfast.org).

A circular mesh, having a radius of 12.5 mm is used having 2030 nodes corresponding to 3901 linear triangular elements. The model is a spectral model, consisting of a concentration of oxy-hemoglobin and deoxy-hemoglobin of 0.01 mM each, water content of 40% with scattering power and density of 1. A bioluminescence source having an arbitrary strength of 10, of radius 2.5 mm was placed at a depth of 7.5 mm (consisting of 75 nodes), with 17 detectors positioned equidistance at the top surface. Each detector point is placed to provide a range of incident angles to normal (with respect to where the camera would be placed) of +/− 10 degrees. Simulations were performed at 4 wavelengths of 590, 610, 630 and 650 nm corresponding to the typical emission of a bioluminescence source.

In order to simulate the effect of angular offset (*n* in Eq. (3), due to geometry of the object, shape and camera location perspective), an offset was added to the data which is a function of the incident angle of the detector to the central axis, i.e. the angle with respect to the camera, which is as it was placed directly above the model. The added offset follows exactly the relationship demonstrated in the experimental measurements, as shown in Fig. 2, namely as a function of the cosine of the incident angle. White Gaussian noise was also added to the modelled data at 1% and 2% of the maximum signal.

**Fig. 3:**
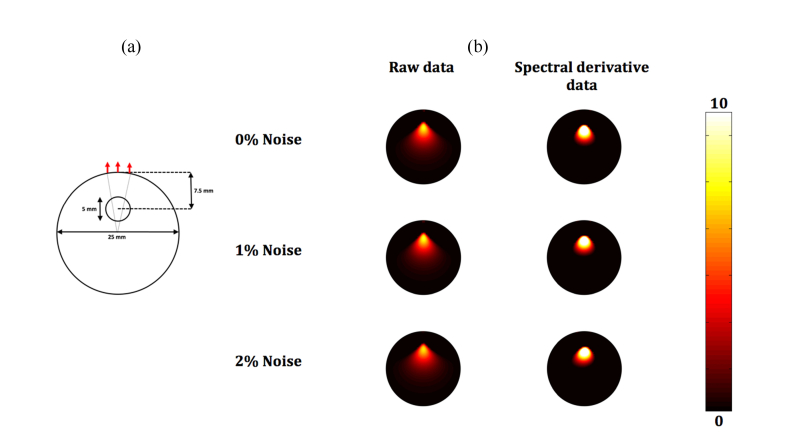
(a) Schematic of 2D circular model with a single bioluminescence source and 17 detectors placed equidistance at +/− 10 degrees from central axis with the camera being placed directly above and (b) Reconstructed images of simulated radial offset added data from 2D circle using ‘raw’ intensity data and ‘logarithm of intensity’ data for different levels of noise.

Images were reconstructed using the noise-added and corrupted data using the conventional approach (raw data) (Eq. (3)) as well as the spectral derivative approach using the logarithm of data (Eq. (8)) and are shown in Fig. 3

.

In order to better quantify the accuracy of each recovery algorithm, the total recovered bioluminescence was also calculated and is shown in Table 1

**Table 1:** The total expected and recovered bioluminescence intensity (AU) using different reconstruction algorithms

	Actual	Conventional	Spectral derivative
0% white noise	750	1062	779
1% white noise	750	1039	759
2% white noise	750	1095	761

. As evident, all the reconstructions using the proposed spectral derivative method is within 4% of the target intensity, whereas using the conventional methods, the total intensity is over-estimated by up to 49%.

### 2.5 Experimental validation

To demonstrate the application of the proposed spectral derivative method on experimental data, a mouse phantom (XFM-2, Perkin Elmer Inc., Waltham, MA, USA) embedded with a self-illuminated rectangular source of dimensions 9.8 mm x 2.8 mm x 2 mm (Trigalight, Mb-Microtec, Niederwangen, Switzerland) was imaged in the BLT system designed for small animal radiation research platform (SARRP) [21]. Multi-spectral and multi-projection bioluminescent images were acquired using filters and a rotating 3-mirror system in the optical system. The mirror can rotate 180 degrees around imaged object, from −90 to + 90 degrees, and reflect the bioluminescence signal to charge-coupled device (CCD) camera (iKon-L 936; Andor Technology, Belfast, UK). BLIs were acquired with 20 nm wide bandpass filters (Chroma Technology Corp, Bellows Falls, VT, USA) at 590, 610, 630 and 650 nm at 3 projections (−90, 0 and + 90 degrees). BLIs were acquired first at 4 pixel binning (~0.4 mm physical size at imaging plane) and exposure time of 1 second. After optical imaging, the phantom along with mouse bed was transferred to the small-animal radiation research platform (SARRP) for cone beam CT (CBCT) imaging. The phantom CBCT was then used to generate the tetrahedral mesh for BLT reconstruction. Since the SARRP acquired CBCT image defines the coordinate used for BLT reconstruction, multi-projection and multi-spectral 2D BLIs need to be mapped to the animal surface of CBCT image. This was achieved using a geometry calibration method to map the 2D optical images acquired at multiple viewing planes onto the animal surface of the 3D CBCT image based on pinhole camera model [21]. This method includes two steps, mapping the CBCT coordinate to the 3D optical coordinate and then projecting the 3D optical coordinate to the 2D optical imaging plane. Once the 3D CBCT and 2D optical coordinates are registered, for a given projection, one can map the surface optical image to the animal surface of the 3D CBCT image. As the data mapping process requires the knowledge of the system geometrical parameters, image markers were placed on the mouse bed for geometrical calibration from which the marker positions can be located in both CBCT and 2D optical images. An optimization routine was then used to retrieve the geometrical parameters by minimizing the difference between the measured and calculated marker positions in the 2D optical coordinate. The average mapping accuracy is within 0.3 mm per marker point. This geometry calibration was performed for every run of the BLT reconstruction to ensure high accuracy of surface data mapping. At the overlapped region between the two mapped projections, for a given surface node, the maximal value between the two projections was chosen as the detector value and data larger than 10% of the maximum value among all the surface data were used for BLT reconstruction.

**Fig. 4:**
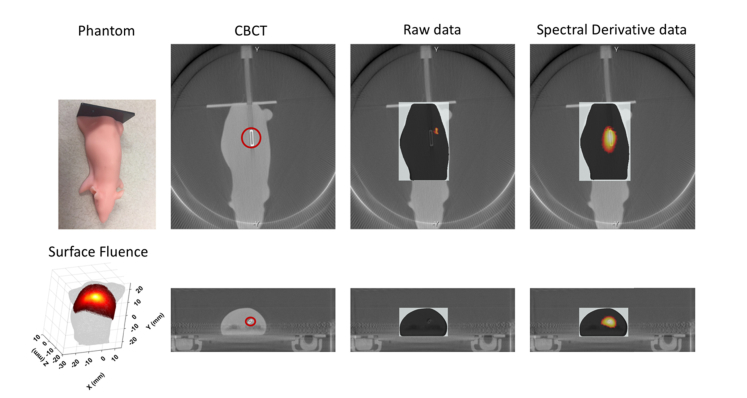
(left top): Photo of the mouse phantom used in the experiment; (left bottom): 3D surface image of the mapped boundary data at 630 nm; (top row): The coronal and (bottom row): transverse slices of the CBCT image, with overlaid recovered maps using the raw as well as spectral derivative data. The red circles shown mark the light source.

The mouse phantom, surface image of the mapped boundary data, and images reconstructed using both the conventional raw data and spectral derivative method are shown in Fig. 4

. The reconstructed image using the ‘raw’ data has recovered a single high intensity source at approximately the correct coronal slice, but it is absent in the transverse slice, whereas with the spectral derivative data, its location has been recovered accurately, as observed in both views. The mesh used for image reconstruction consisted of 31611 nodes, corresponding to 175518 linear tetrahedral elements.

## 3. Discussions

The position and shape of an object being imaged in a non-contact BLI system can greatly affect the accuracy of the detected fluence from the boundary of the object. It is already understood that the attenuation of the bioluminescence signal is affected by the underlying spectrally varying optical properties of biological tissue, however as the light exiting the boundary of a diffusive object is Lambertian in nature, it is shown that the intensity and distribution of light from a tissue mimicking phantom varies as the object rotates with respect to the imaging camera, Fig. 1. This variation in intensity is shown to be as much as 23% when the phantom is rotated by 60 degrees and 41% at 90 degrees, Fig. 2(a). Given that in most pre-clinical studies, it is common practice to associate the total count in intensity being proportional to the total amount of activity (for example total number of tagged cells), a small variation in animal positioning can lead to a large variation in detected activity which may be significant.

To demonstrate that this variation in detected intensity with angular rotation is due to Lambert's cosine law (i.e. it is Lambertian in nature), the maximum detected intensity at each angular rotation is plotted against cosine of the turning angle in Fig. 2(b). This demonstrates the importance of accounting for the surface geometry in non-contact 3D imaging, as often utilized by commercial systems, to allow for detected signal variation due to the Lambertian emission in BLI. This effect has been known, but often ignored with most recently, work being published that outlines the use of a body conforming animal mold together with automated analysis tools to account for such variations [22]. Other works to date have also dealt with this issue using free-space models both in non-contact Fluorescence imaging [23] as well as BLT [17].

The significance of this variation of measured intensity as a function of camera properties and animal shape/geometry has been demonstrated using both a simplified 2D model, Fig. 3, as well as experimental data from a mouse shaped phantom, Fig. 4. In both cases, it is demonstrated that the utilization of raw data which is not calibrated to account for these variations, using conventional algorithms fails to provide an accurate map of the internal bioluminescence source distribution. To overcome this, it is possible to employ a free-space model as outlined previously [17], but this can often be time consuming and complex due to the need to accurately model paths of photons from each surface of the animal onto the measurement device pixel and to accurately calculate the inverse of its associated transfer function [17, 24]. Therefore, there is a need to develop image reconstruction algorithms that are independent of such errors in data to further improve accuracy of recovered bioluminescence source recovery without the need of complex and intensive computation.

The use of derivative data in diffuse optical imaging and spectroscopy is commonplace, where the derivative data can be either in spectral [20, 25] or temporal [26, 27] and these are used to account for unknown factors within the measured data. In all cases of diffuse optical imaging and spectroscopy, the data utilized for parameter recovery has been transformed from ‘raw intensity’ data to ‘logarithm of intensity’ to account for the large dynamic range of measurements, whereas image reconstruction in BLT has always used ‘raw intensity’ data. In order to implement a spectral derivative algorithm for BLT, a data transformation is presented in Eq. (4), which when utilized across neighboring wavelengths allows for the cancellation of the angular dependent variations discussed above, Eq. (8). The proposed algorithm is tested using both the simplified 2D model, Fig. 3, as well as experimental data from a phantom, Fig. 4. In both cases, it is demonstrated that the utilization of spectral derivative data significantly improves the accuracy of calculated spatial map of the internal bioluminescence source distribution. The data with simulated models has shown to be robust to errors due to the angular dependent measurement errors, reducing the quantitative error for the reconstructed source intensity from 49% to 4%. Using experimental data, although the true value of the intensity is not known, qualitatively the improved accuracy of the reconstructed source is apparent in Fig. 4.

The proposed algorithm in this work does not require additional data collection and does not rely on any modification to most non-contact optical imaging systems. The normalization of the measured data with respect to its logarithm are shown to be simple and do not require intensive computation. This should be valid for any combination of wavelengths as long as the assumption that the light-path between the domain being imaged and the detection camera is correctly assumed to be constant. It is shown here that such modification to measured data to allow the utilization of spectral derivative measurements are beneficial and although known for their application to diffuse optical and Fluorescence tomography, have not been reported or considered for Bioluminescence imaging.

## 4. Conclusions

This work highlights the importance of accounting for both the imaging domain’s shape and relative position with respect to the measurement device in non-contact Bioluminescence Imaging and Tomography. It is shown that the measured intensity from a non-contact system, due to an internal light source can vary by as much as 41% due to rotational variation of 60 degrees. A new 3D image reconstruction algorithm is presented, whereby instead of utilizing raw spectral intensity data, the spectral derivative of the logarithm of data is incorporated to account for errors in data which are spectrally independent. The basis of the algorithm has been presented together with data from a mouse shaped phantom study to demonstrate its accuracy and effectiveness. This proposed algorithm is easily adaptable to all commercial systems and does not require any additional hardware calibration, design or implementation.
